# Antioxidant efficacy and the upregulation of Nrf2-mediated HO-1 expression by (+)-lariciresinol, a lignan isolated from *Rubia philippinensis*, through the activation of p38

**DOI:** 10.1038/srep46035

**Published:** 2017-04-05

**Authors:** Vivek K. Bajpai, Md Badrul Alam, Khong Trong Quan, Kyoo-Ri Kwon, Mi-Kyoung Ju, Hee-Jeong Choi, Jong Sung Lee, Jung-In Yoon, Rajib Majumder, Irfan A. Rather, Kangmin Kim, Sang-Han Lee, MinKyun Na

**Affiliations:** 1 Department of Applied Microbiology and Biotechnology, School of Biotechnology, Yeungnam University, Gyeongsan, Gyeongbuk 38541, Korea; 2 Department of Food Science and Biotechnology, Graduate School, Kyungpook National University, Daegu 41566, Korea; 3 College of Pharmacy, Chungnam National University, Daejeon 34134, Korea; 4 Kcellbio, Seoulsoop Kolon Digital Tower, Seongsuil-ro-4-gil, Seongdong-gu, Seoul, 04713, Korea; 5 Department of Biological Sciences, Macquarie University, Sydney, NSW 2109, Australia; 6 Elizabeth Macarthur Agricultural Institute (EMAI), NSW Department of Primary Industries, Menangle, NSW 2567, Australia; 7 Division of Biotechnology, College of Environmental and Bioresource Sciences, Chonbuk National University, 79 Gobong-ro, Iksan-si-570-752, Jeonbuk, Republic of Korea

## Abstract

The aim of the present study was to examine the antioxidative activity of (+)-lariciresinol (LRSL), an optically active lignan isolated from *Rubia philippinensis* in several *in vitro* assays. LRSL was also subjected to evaluate its inhibitory effect against the generation of reactive oxygen species (ROS) in murine macrophage (RAW 264.7) cells. The results showed that LRSL possessed very strong radical scavenging activity and reducing power, as well as inhibited ROS generation in a dose-dependent manner without showing any cytotoxicity. The transcriptional and translational levels of superoxide dismutase (SOD), glutathione peroxidase (GPx) and catalase (CAT) were markedly higher in the sample treated group. LRSL treatment also increased the transcriptional and translational activities of NF-E2-related factor-2 (Nrf-2) with a corresponding increase in the transcriptional and translational activities of the heme oxygenase-1 (HO-1). LRSL activated p38 and treatments with SB239063 (a p38 inhibitor) suppressed the LRSL-induced activation of Nrf2, resulting in a decrease in HO-1 expression. Collectively, the data demonstrated that LRSL has potent antioxidative activity, decreasing ROS generation in RAW 264.7 cells and increasing the transcriptional and translational levels of antioxidant enzymes by activating Nrf2-mediated HO-1 induction via p38 signaling.

Toxic reactive oxygen species (ROS) are generated during aerobic metabolism, and excessive generation of ROS, which include superoxide anions (O_2_•−), hydrogen peroxide (H_2_O_2_), and hydroxyl radicals (OH•), causes oxidative stress^1^. Oxidative stress plays a pivotal role in the pathogenesis of various chronic diseases, such as diabetes, cancer and cardiovascular disorders, thus, interest has recently been increasing in the use of natural antioxidants for the maintenance of human health and for the prevention and treatment of various chronic diseases^2^. Antioxidants have shown their cellular protection against oxidative stress by direct or indirect pathway depending upon their working mechanism. In direct pathways, antioxidant have been scavenged reactive oxygen and nitrogen species by being consumed or chemically modified^3^. In contrast, indirect pathways have been involved with upregulating phase II detoxifying and antioxidant enzymes^3^.

Aerobic organisms have effective antioxidant networks to defend against oxidative stress, involving primary enzymes such as superoxide dismutase (SOD), glutathione peroxidase (GPx) and catalase (CAT) as well as inducible phase II detoxifying enzyme such as heme oxygenase-1 (HO-1), NAD(P)H dehydrogenase [quinone] 1 (NQO1), Glutamate-cysteine ligase catalytic subunit (GCLc), Glutamate-cysteine ligase regulatory subunit (GCLm) through the activation of nuclear transcription factor-erythroid 2 related factor (Nrf2)^4^. Under physiological conditions, Nrf2 is sequestered by binding to Kelch-like ECH-associated protein 1 (Keap1), which inhibits the translocation of Nrf2 into the nucleus. The conformational change of Keap1 through interaction with different types of inducers release the Nrf2, translocates to the nucleus and binds to antioxidant-related elements in the promoter regions of antioxidant and cytoprotective genes^5^. In addition, several signaling cascades such as mitogen-activated protein kinase (MAPK), phosphatidylinositol 3-kinase (PI3K/Akt), protein kinase C (PKC), signaling pathways are involved in phosphorylation of Nrf2 as well as translocate into nucleus^6^,^7^.

(+)-Lariciresinol, an optically active lignan ([α]_D_+17 (*c* 0.05, MeOH)) isolated for the first time from *Rubia philippinensis*, has a dimeric structure of a phenylpropanoid with a core structure of tetrahydrofurano ring. Both (+)-lariciresinol and (−)-lariciresinol have been found in nature, but they have different physicochemical property^8^,^9^ and biological activities^10^,^11^. Antioxidant properties of (+)-lariciresinol on lipid peroxidation^12^, peroxy radical^13^, and superoxide radical^14^ were demonstrated previously. The antioxidant activity of (+)-lariciresinol was equivalent to that of secoisolariciresinol and higher than that of other tetrahydrofurano lignans, matairesinol, hydroxylmatairesinol, and nortrachelogenin in Trolox equivalent antioxidant capacity test^15^. However, the underlying mechanism, by which (+)- lariciresinol mitigate the oxidative stress, remains unclear.

We therefore hypothesized that (+)-lariciresinol (LRSL) functionally associates with Nrf2 to stimulate the expression of certain antioxidant enzymes. In the present study, we focused on the regulatory role of LRSL in antioxidant capacity as measured the various *in vitro* antioxidant assays, and the expression level of antioxidant enzymes in RAW 264.7 cells. We also elucidated the mechanisms by measuring the activation of Nrf2 function by LRSL in RAW 264.7 cells. The results demonstrated that LRSL regulates antioxidant enzymes by modulating Nrf2 function via activation of the p38 pathway in RAW 264.7 cells.

## Materials and Methods

### Plant materials

The roots of *Rubia philippinensis* were collected from Bidoup-Nui Ba National Park, Lamdong province, Vietnam in July 2013, and identified by Dr. Phuong Thien Thuong at the Department of Pharmaceutical Analysis and Herbal Standardization, National Institute of Medicinal Materials, Vietnam. A voucher specimen was deposited at the herbarium of the National Institute of Medicinal Materials, Hanoi, Vietnam, as well as the Laboratory of Pharmacognosy at the College of Pharmacy, Chungnam National University, Daejeon, Korea.

### Extraction, isolation, and characterization of (+)-lariciresinol

(+)-Lariciresinol was obtained from the roots of *R. philippinensis* by chromatographic methods. Briefly, the ethanol extract of *R. philippinensis* (150 g) was suspended in H_2_O (1.5 L) and partitioned with CH_2_Cl_2_ (2 L × 3) to yield CH_2_Cl_2_ extract. The CH_2_Cl_2_-soluble fraction (50 g) was subjected to silica gel VLC and eluted with *n*-hexane-EtOAc (20:1, 10:1, 5:1, 3:1, 2:1) and CHCl_3_-MeOH (8:1) to afford six fractions (D-1 → D-6). Fraction D-6 (10 g) was divided into 11 sub-fractions (D-6-1 → D-6-11) using MPLC with a gradient of MeOH-H_2_O (10:90 → 100:0, 7 L). (+)-Lariciresinol (*t*_*R*_ 33.0 min, 31 mg) was obtained from D-6-3 (360 mg) by HPLC eluting with MeOH-H_2_O (45:55, 4 mL/min, UV 254 nm).

(+)-Lariciresinol: brownish amorphous powder, [α]_D_+17 (*c* 0.05, MeOH), ^1^H NMR (300 MHz, methanol-*d*_4_): 2.38 (1 H, m, H-8), 2.47 (1 H, dd, *J* = 13.1, 11.7, H_a_-7′), 2.72 (1 H, m, H-8′), 2.91 (1 H, dd, *J* = 13.1, 4.5, H_b_-7′), 3.63 (1 H, dd, *J* = 10.8, 6.6, H_a_-9), 3.72 (1 H, dd, *J* = 8.1, 6.1, H_a_-9′), 3.82 (1 H, overlapped, H_b_-9), 3.97 (1 H, dd, *J* = 8.1, 6.1, H_b_-9′), 4.75 (1 H, d, *J* = 6.9, H-7), 6.63 (1 H, dd, *J* = 8.0, 1.2, H-6′), 6.73 (1 H, d, *J* = 8.0, H-5′), 6.78 (3 H, overlapped, H-6, H-5, H-2′), 6.91 (1 H, d, *J* = 1.2, H-2); ^13^C NMR (75 MHz, methanol-*d*_4_): 135.7 (C-1), 110.6 (C-2), 148.9 (C-3), 146.9 (C-4), 116.0 (C-5), 119.8 (C-6), 83.9 (C-7), 53.9 (C-8), 60.4 (C-9), 133.5 (C-1′), 113.4 (C-2′), 148.9 (C-3′), 145.7 (C-4′), 116.2 (C-5′), 122.1 (C-6′), 33.6 (C-7′), 43.8 (C-8′), 73.4 (C-9′), 56.3, 56.3 (2 × OCH_3_-3,3′) (Figs S1–S3).

### Drugs and chemicals

2,2-diphenyl-1-picrylhydrazyl (DPPH), 2,2′-Azobis(2-amidinopropane) dihydrochloride (AAPH), phenazine methosulfate (PMS), neocaprione, 2,4,6-Tris(2-pyridyl)-s-triazine (TPTZ), 3-(4,5-dimethylthiazol-2-yl)-2,5-diphenyltetrazolium bromide (MTT), 2′,7′-Dichlorofluorescin diacetate (DCFH-DA), phosphate buffered saline (PBS, pH 7.4), and dimethylsulfoxide (DMSO) were purchased from Sigma Aldrich (St. Louis, MO, USA). DMEM (Dulbecco’s modified Eagle’s medium), fetal bovine serum (FBS), penicillin-streptomycin mixture, 0.25% trypsin-EDTA, were purchased from Gibco-BRL Life Technologies (Grand Island, NY, USA). Antibodies purchased from Santa Cruz Biotechnology (Santa Cruz, CA, USA) included anti-SOD1, anti-HO-1, anti-catalase (CAT), anti-Glutathione peroxidase 1 (GPx-1), anti-Nrf2, whereas anti-phospho-JNK, Anti-p44/42MAPK (ERK1/2), and anti-phospho-p44/42MAPK (ERK1/2), anti-phospho-p38, Anti-p38 were purchased from Cell Signaling Technology (Beverly, MA, USA).

### Radical-scavenging activity assays

2,2-Diphenyl-1-picrylhydrazyl (DPPH) radical-scavenging assay was used for the evaluation of the free radical scavenging activity of LRSL and was conducted following the protocol described elsewhere^16^, with a minor modification. Briefly, a 0.2 mM solution of DPPH in 50% ethanol was used and 198 μl of this solution was added to 2 μl of various concentrations of the LRSL. The mixture was allowed to stand at room temperature for 10 min and the absorbance was measured at 517 nm in a multi-label counter (Victor3, PerkinElmer, Waltham, MA, USA, USA). Ascorbic acid was tested as a standard antioxidant compound. The percent inhibition ability was calculated using the following equation:


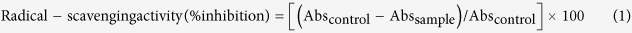


Where Abs_control_ is the absorbance of the control and Abs_sample_ is the absorbance of the sample. All samples were analyzed in triplicate.

The method of Re *et al*.^17^ was adopted for the ABTS assay with slight modification. A 2 μl of varying concentrations of the LRSL were allowed to react with 198 μl of the ABTS ^•+^ solution and the absorbance readings were recorded at 734 nm. Ascorbic acid was tested as a standard antioxidant compound. The percent inhibition ability was calculated using the equation 1.

Superoxide radical (SOD) was generated by non-enzymatic phenazine methosulfate-nicotinamide adenine dinucleotide (PMS/NADH) system, which reduces NBT to a purple color formazan and the scavenging activity of SOD by LRSL was measured according to the previously reported method with minor modification^18^. Gallic acid was used as a positive control and the percent inhibition ability was calculated using the equation 1.

A previously described method was adopted for determining the hydroxyl radical (OH), scavenging activity of LRSL with minor modification for which hydroxyl ions were generated by the Fenton reaction using Fe^3+^-ascorbate-EDTA-H_2_O_2_ system^19^. The assay is based on the quantification of the degradation product of 2-deoxy-2-ribose sugar by condensation with 2-thiobarbituric acid (TBA) to form pink color, which was measured at 532 nm and the equation 1 was used to determine the percent inhibition ability of LRSL.

For the measurement of reducing power, the ferric reducing antioxidant power (FRAP) assay was carried out, as described previously with a slight modification^20^. Ascorbic acid was also tested as a standard antioxidant compound and ascorbic acid equivalent FRAP value (μM) was calculated from the standard curve of ascorbic acid.

Cupric-reducing antioxidant capacity (CUPRAC) of LRSL was determined according to the method^21^, with slight modifications and ascorbic acid equivalent CUPRAC value (μM) was calculated from the standard curve of ascorbic acid.

The oxygen radical absorbance capacity (ORAC) assay was carried out according to a previous report^22^. Trolox, a water-soluble analogue of vitamin E, was used as a positive control. The experiment was conducted at 37 °C under pH 7.4 conditions with a blank sample in parallel. The analyzer was programmed to record the fluorescence of 200 nM fluorescein every minute, after addition of 20 mM AAPH with a 480 nm excitation and a 520 nm emission wavelength. The results were calculated using the differences in the areas under the fluorescence decay curves between the blank sample and experimental sample and were expressed as area under the curve (Net AUC) values.

### Cell culture and cell viability assay

RAW 264.7 cells (ATCC, Rockville, MD, USA) were cultured at 37 °C in DMEM supplemented with 10% FBS, streptomycin–penicillin (100 μg/ml each; Hyclone) in a humidified atmosphere of 5% CO_2_. The tetrazolium dye colorimetric test (MTT) was used to determine the viability of RAW 264.7 cells. RAW 264.7 cells were first cultured in 96-well plates (5 × 10^5^ cells/well) for 24 h, and treated with different concentrations of LRSL. After 24 h incubation, MTT reagent was added to each well and the plate was incubated at 37 °C for 1 h. The medium was removed and the plate was washed twice with PBS (pH 7.4). The intracellular insoluble formazan was dissolved in 100% DMSO. The absorbance of each cell was then measured at 570 nm using a microplate reader (Victor3, PerkinElmer, Waltham, MA, USA) and the percentage viability was calculated.

### Measurement of intracellular ROS

Cellular oxidative stress due to reactive oxygen species (ROS) generated by AAPH was measured spectrofluorometrically using the DCFH-DA method^23^. RAW 264.7 cells were first cultured in 96-well plates (5 × 10^5^/mL) with DMEM for 24 h. The cells were pretreated with various concentration LRSL. After 1 h, cells were stimulated with AAPH (600 μM) and incubated for an additional 30 min. After washing with phosphate-buffered saline (PBS) twice, the cells were treated with 25 μM DCF-DA for 30 min at 37 °C. The fluorescence intensity was measured at an excitation wavelength of 485 nm and emission wavelength of 528 nm using a fluorescence microplate reader (Victor3, PerkinElmer, Waltham, MA, USA).

### Cytosolic and nuclear protein fractionation

After the LRSL treatments at indicated time and concentration, cultures were harvested and cells pelleted by centrifugation at 280 g for 10 min, and washed twice with 1x PBS. Cytosolic and nuclear proteins were extracted from the freshly harvested cells using a commercially available CelLyticTM NuCLERTM extraction kit (Sigma, St. Louis, MO, USA). Briefly, the washed cell pellets were gently resuspended in hypotonic lysis buffer consisting of 10 mM HEPES (pH 7.9), 10 mM KCl, 1.5 mM MgCl_2_, 1 mM DTT, and 1x protease inhibitor cocktail. After incubation on ice for 15 min to allow cells to swell, NP-40 detergent was added to 0.25% and the sample was vigorously vortexed for 10 sec to disrupt cell membrane. Centrifugation at 10,000 g for 30 seconds separated the cytosolic fraction (supernatant) from the nuclei-enriched fraction (pellet). The cytosolic fraction was stored at −80 °C. The nuclear fraction was washed twice with the hypotonic lysis buffer using the same centrifugation protocol then nuclear proteins were extracted from the nuclei by a hypertonic buffer (20 mM HEPES pH 7.9, 1.5 mM MgCl2, 0.42 mM NaCl, 25% [v/v] glycerol, 1 mM DTT, and 1x protease inhibitor cocktail) through vigorous agitation for 20 min at room temperature (RT), centrifuged at 16,000 g for 10 min. The final supernatant (nuclear extract) was collected for storage at −80 °C. The specificity of this subcellular fraction method was supported by immunoblotting.

### Reverse Transcription-polymerase chain reaction (RT-PCR)

Total RNA was extracted using TRI-zol (Invitrogen Co., Carlsbad, CA, USA) according to the manufacturer’s instructions. Total RNA (2 μg) was reverse transcribed using reverse transcriptase (MP Biomedicals, Santa Ana, CA, USA) and oligo(dT) primers. cDNA was amplified using a PCR Thermal Cycler Dice TP600 (Takara Bio Inc., Otsu, Shiga, Japan). PCR products were visualized by ethidium bromide staining after electrophoresis. The bands were analyzed using the Image Lab™ Software, version 5.2.1 (Bio-Rad laboratories, CA, USA). Specific oligonucleotide primers for mouse transcripts were used (Table 1).

### Preparation of cell lysates and western blotting

RAW 264.7 cell lysates were prepared using a standard protocol, mixed with sample buffer (250 mM Tris-HCl (pH 6.8), 0.5 M DTT, 10% SDS, 0.5% bromophenol blue, 50% glycerol, 5% 2-mercaptoethanol), and denatured at 100 °C for 5 min. For nuclear protein extraction, a nuclear/cytosolic fractionation kit (Cell Biolabs, Inc., San Diego, CA, USA) was used. Sample proteins (20 μg) were separated by 10% SDS-PAGE. Following electrotransfer to nitrocellulose membranes (Whatman, Dassel, Germany), the membranes were incubated overnight with primary antibody in 5% skim milk. Anti-SOD1, anti-HO-1, anti-catalase (CAT), anti-glutathione peroxidase 1 (GPx-1), anti-Nrf2, and β-actin antibodies (Santa Cruz Biotechnology, Inc., Santa Cruz, CA, USA) were used as primary antibodies. Anti-goat IgG-horse radish peroxidase (HRP) (Santa Cruz) and anti-rabbit IgG-HRP (Santa Cruz) were used as secondary antibodies. The antigen-antibody reaction was detected using an ECL solution system (Perkin Elmer). The bands were analyzed using the Image Lab™ Software, version 5.2.1 (Bio-Rad laboratories, CA, USA).

### Statistical analysis

All data are expressed as the mean ± SD. Data were analyzed using one-way ANOVA. Differences were considered significant of p < 0.05. All analyses were performed using SPSS for Windows, version 10.07 (SPSS, Chicago, IL, USA).

## Results

### Identification and characterization of (+)-lariciresinol

The ^1^H NMR data of purified compound, [α]_D_+17 (*c* 0.05, MeOH), displayed two pairs of aromatic ring systems at δ_H_ 6.63 (1 H, dd, *J* = 8.0, 1.2, H-6′), 6.73 (1 H, d, *J* = 8.0, H-5′), 6.78 (3 H, overlapped, H-6, H-5, H-2′), and 6.91 (1 H, d, *J* = 1.2, H-2). In addition, an oxymethylene moiety at δ_H_ 3.72 (1 H, dd, *J* = 8.1, 6.1, Ha-9′), 3.97 (1 H, dd, *J* = 8.1, 6.1, Hb-9′), two methine groups at δ_H_ 2.38 (1 H, m, H-8) and 2.72 (1 H, m, H-8′), and an oxymethine at δ_H_ 4.75 (1 H, d, *J* = 6.9, H-7) indicating a tetrahydrofuran substructure were observed in the ^1^H NMR data as well. The ^13^C NMR spectrum showed 18 signals for a typical lignan derivative. On the basis of NMR spectroscopic data analyses and specific rotation, the compound was identified as (+)-lariciresinol^8^.

### Radical scavenging activities of (+)-lariciresinol (LRSL)

**Figure 1:**
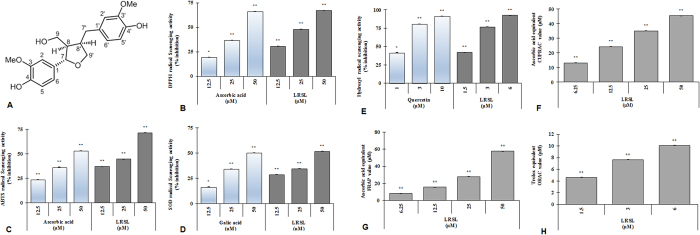
Chemical structure and radical scavenging effects of (+)-lariciresinol. The chemical structure of (+)-Lariciresinol (**A**). The DPPH radical scavenging assay (**B**), ABTS ^•+^ radical scavenging assay (**C**), superoxide (SOD) radical scavenging assay (**D**), hydroxyl (HO) radical scavenging assay (**E**), CUPRAC assay (**F**), and FRAP assay (**G**) were conducted with various concentrations of LRSL, whereas ascorbic acid, gallic acid and quercetin were tested as standard antioxidant compounds. The ORAC activities (**H**) of the samples were calculated by subtracting the area under the blank curve from the area under the sample curve, to obtain the net area under the curve (Net AUC). *p < 0.05 and **p < 0.01, significantly different from control, using student’s t-test.

Antioxidants protect cellular damage from oxidative stress by direct and indirect ways depending upon the type of working mechanism^3^. Direct antioxidant capacity may defined as to scavenge free radical, reactive oxygen and nitrogen species by donating hydrogen or electrons. In contrast, the indirect antioxidant capacity is involved to mitigate the oxidative stress *via* the expression of phase II detoxifying and antioxidant genes^3^. To investigate whether (+)-lariciresinol (LRSL) have the direct antioxidant potential in respect of radical scavenging activities, DPPH-, ABTS-, superoxide-, and hydroxyl- radical scavenging activities were performed. LRSL significantly scavenged the DPPH^•^, a stable organic nitrogen radical, in a dose dependent manner (Fig. 1B). Furthermore, in the reduction of the radical cation from 2,2′-azinobis-(3-ethylbenzothiazoline)-6-sulfonic acid (ABTS ^•+^), a mixed electron transfer and hydrogen atom transfer assay, LRSL dose dependently reduced the ABTS ^•+^ (Fig. 1C). The superoxide (SOD) radical scavenging ability of LRSL was assessed by the PMS-NADH superoxide generating system and the results were shown in Fig. 1D. Hydroxyl radicals are extremely reactive free radicals formed in biological systems and have been capable of damaging almost every molecule found in living cells. In this study, Fig. 1E showed that LRSL strongly inhibited the production of hydroxyl radical from the Fenton reaction in a concentration dependent manner. Additionally, whether LRSL have electron donating potentiality, we assessed cupric-reducing antioxidant capacity (CUPRAC), ferric reducing antioxidant power (FRAP) and oxygen radical absorbance capacity (ORAC) and found that LRSL have strong reducing power capacity in a concentration dependent manner (Fig. 1F–H). Based on these observations, we speculated that LRSL have a very strong capacity to scavenge various free radicals through hydrogen atom transfer/electron donation.

### (+)-Lariciresinol attenuates cellular oxidative stress induced by AAPH (*2,2*′*-Azobis(2-amidinopropane)dihydrochloride*) in RAW 264.7 cells

**Figure 2:**
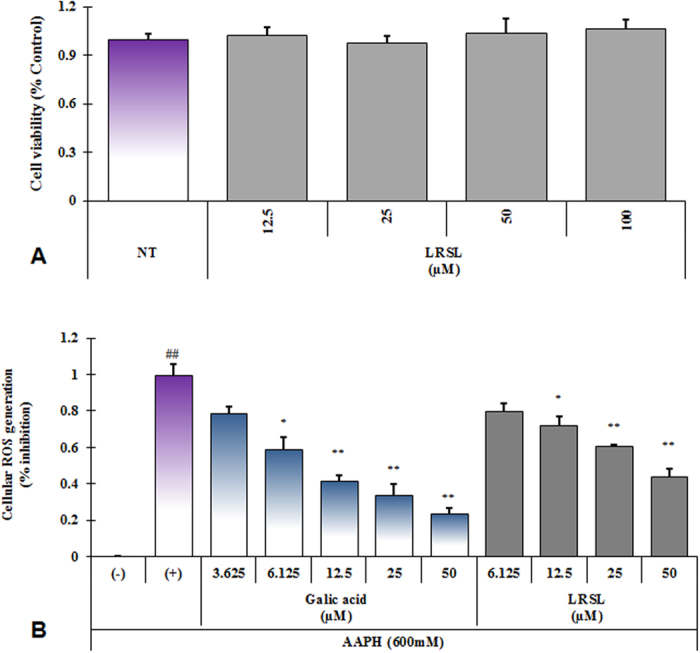
Cell viability and inhibition of ROS generation in RAW 264.7 cells. RAW 264.7 cells were seeded at a density of 2 × 10^4^ cells per well (96-well plate) and MTT assay (**A**), and DCFH-DA assay (**B**) were conducted with various concentrations of LRSL. ##p < 0.001, significantly different from normal control, *p < 0.05 and **p < 0.01 significantly different from AAPH treatment, using student’s t-test.

As LRSL provoked strong radical scavenging activities, subsequently, the cellular ROS scavenging activity, induced by AAPH, which is an extensively reported generator of free radicals, of LRSL was performed. In this assay, gallic acid, a known standard antioxidant, was used as a positive control. Water soluble AAPH can easily produce one mole of nitrogen and two moles of carbon radicals, which could generate peroxyl radicals by combining with molecular oxygen and maintaining a constant rate of free radical production in the solution^24^. It was observed in this assay that LRSL treatment inhibited the generation of ROS in AAPH-induced RAW 264.7 cells in a dose-dependent manner (Fig. 2B) without any cellular toxicity (Fig. 2A).

### Effects of (+)-lariciresinol on antioxidant enzyme expression in RAW 264.7 cells

**Figure 3:**
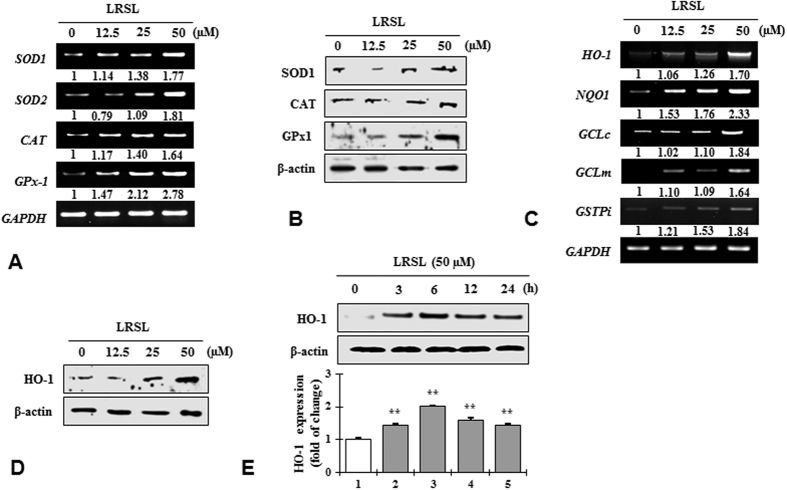
Analysis of primary and phase II antioxidant and detoxifying enzyme. RAW 264.7 cells were pretreated for 24 h with specified concentrations of LRSL. The mRNA expressions of primary antioxidant enzyme (**A**), phase II antioxidant and detoxifying enzymes (**C**) were measured by RT-PCR. The protein levels of primary antioxidant enzyme (**B**), and HO-1 in concentration (**D**), and time-dependent (**E**) were determined by western blot.

To investigate the effects of LRSL on antioxidant enzymes (SOD1, CAT and GPx-1) and phase II detoxifying enzymes (HO-1, NQO1, GCLc and GCLm), RAW 264.7 cells were treated with LRSL at 12.5, 25 and 50 μM for 24 h. RT-PCR analysis indicated a dose-dependent increase in the mRNA expression of SOD1, CAT and GPx-1 (Fig. 3A) and phase II detoxifying enzymes HO-1, NQO1, GCLc and GCLm (Fig. 3C). The protein levels of SOD1, CAT, GPx-1 (Fig. 3B) and HO-1 (Fig. 3D) were further confirmed by western blot analysis. In addition, time-dependent western blot analysis revealed significantly enhanced protein expression of HO-1, from 3 h to 24 h, peaking at 6 h after LRSL treatment. (Fig. 3E). These data suggested that the antioxidant activity of LRSL might be related to activate primary antioxidant enzymes as well as phase II detoxifying enzymes through increasing their expression at both the mRNA and protein level.

### Effects of (+)-lariciresinol on phase II enzymes through Nrf2 nuclear translocation in RAW 264.7 cells

**Figure 4:**
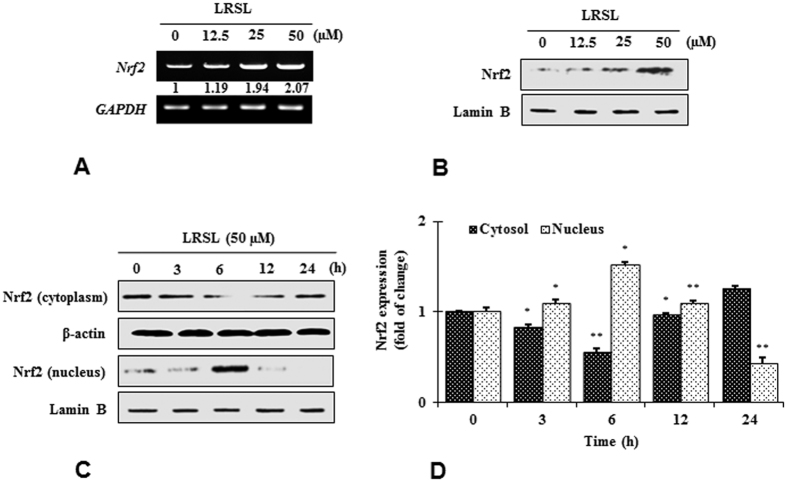
Effect of LRSL on translocation of Nrf2. RAW 264.7 cells were pretreated for 24 h with specified concentrations of LRSL. The mRNA expression of Nrf2 (**A**), was measured by RT-PCR. The protein level of nuclear Nrf2 in concentration (**B**), and time-dependent (**C**,**D**) manner was determined by western blot.

**Figure 5:**
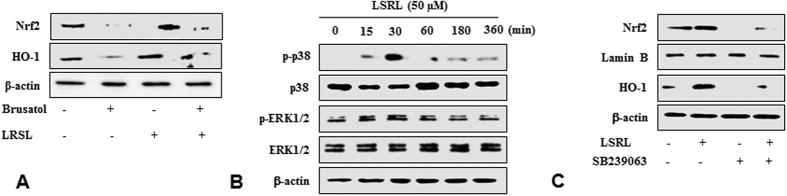
LRSL activates the translocation of Nrf2 through the activation of p38. RAW 264.7 cells were pretreated with LRSL and Nrf2 inhibitor (Brusatol). Nrf2 and HO-1 expressions were analyzed by western blotting (**A**), RAW 264.7 cells were pretreated with LRSL for the indicated time and kinase activation was analyzed by western blotting (**B**). Cells were treated with specific inhibitor SB239063 and the protein levels of Nrf2 and HO-1 were analyzed by western blotting (**C**).

Under normal conditions, Nrf2 is generally coupled with the Keap1 protein, and phase II enzyme activation can only be attained when Nrf2 is set free and translocates to the nucleus. Therefore, to clarify the mechanism of Nrf2 activation by LRSL, the mRNA as well as the protein levels of Nrf2 in the nucleus and the cytosol were analyzed. It was noticed that LRSL treatment increased the mRNA level of Nrf2 in a dose dependent manner. (Fig. 4A). As expected, the nuclear Nrf2 content was also markedly increased after LRSL treatment (Fig. 4B). Further, a time course study also revealed that a time dependent increase in Nrf2 translation with a peak level 6 h after LRSL treatment. (Fig. 4C). Based on these observations, it is inferred that LRSL might disrupt the binding of Nrf2 with Keap1, freeing Nrf2 to under nuclear translocation. To confirm that LRSL activates phase II enzymes (HO-1, NQO1, GCLc and GCLm) through Nrf2, brusatol (a specific Nrf2 inhibitor) was treated into cells before LRSL treatment. Brusatol caused significant inhibition of Nrf2 protein levels, which was not affected by the addition of LRSL (Fig. 5A). In addition, the induction of HO-1 protein by LRSL was also efficiently abolished as a result of Nrf2 inhibition (Fig. 5A). The results suggesting that LRSL induced HO-1 upregulation via Nrf2 mediated signaling.

### (+)-lariciresinol activates Nrf2 via phosphorylation of p38

To investigate the pathways involved in the activation of the Nrf2/Keap1 system, RAW 264.7 cells were treated with LRSL for 0.5, 1, 3, and 6 h, and the phosphorylation of JNK, Erk1/2, and p38 was assessed via western blotting. As shown in Fig. 5B, LRSL treatment showed notably enhanced p38 and ERK1/2 phosphorylation after 15 and 30 min. However, the phosphorylated forms of JNK were not detected in LRSL treated cells (Fig. S4). Therefore, to analyze whether phosphorylation of p38 and ERK1/2 pathways was involved in the induction of Nrf2 action and HO-1 expression, the specific inhibitors, such as SB 239063 for p38 and PD187352 for ERK were applied to cells treated with LRSL. As expected, LRSL increased nuclear Nrf2 accumulation and protein expression of HO-1, whereas inhibiting p38 pathways, but not ERK1/2 (data not shown) strongly mitigate that these effects were LRSL mediated (Fig. 5C). These results indicate that only p38 is involved in LRSL induced activation of Nrf2 mediated expression of HO-1 in RAW 264.7 cells.

## Discussion

Oxidative stress is defined as an imbalance between the production of free radicals and their elimination by cellular defense mechanisms. ROS, including superoxide anion (O_2_^•^), hydrogen peroxide (H_2_O_2_) and hydroxyl radical (OH^•^), are by-products of cellular metabolic reactions and pathways and are intracellular, toxic species, partially produced by reduction of oxygen (O_2_)^1^. It is well documented that ROS are chemically more reactive than O_2_^•^, thus, ROS were thought to function exclusively as cellular damaging agents, indiscriminately reacting towards lipids, proteins, and DNA^25^. In this study, the antioxidant effects of LRSL were evaluated using various *in vitro* chemical assays and for the first time, the underlying mechanism by which LRSL mitigates the oxidative stress through the transcriptional and translational regulation of phase I oxidoreductase and phase II detoxifying enzymes via Keap1-Nrf2 pathway in RAW 264.7 cells by activating p38 signaling pathways.

A compound becomes an antioxidant if it (i) has a hydrogen or electron-donating capacity, (ii) has the ability to stabilize and delocalize the unpaired electron, and (iii) has the metal-chelating potential^26^. Thus, the antioxidant activity of biologically active compounds cannot be evaluated by a single method. Hence, to explore and understand these possible mechanisms, several antioxidant assays, including DPPH, ABTS, SOD, and OH-radical scavenging assays as well as FRAP, CUPRAC, and ORAC assays were performed for evaluating the antioxidant activities of LRSL. The results confirmed that LRSL have a broad range of antioxidant properties. The radical scavenging activities of LRSL are summarized in Fig. 1. The DPPH^•^ and ABTS^+•^ are considered the most popular spectrophotometric methods for the determination of the antioxidant capacity of any extracts or compounds. The magnitude of free radical quenching in both DPPH and ABTS assay was dose dependent and steadily increased with increase in sample concentration (Fig. 1B,C). These findings revealed a fact that LRSL have the capacity to scavenge free radical in two different mechanisms, firstly, through a single electron transfer reaction (ABTS assay) and secondly, a hydrogen transfer reaction (DPPH assay)^27^. On the other hand, superoxide anion, a weak oxidant, gives rise to generate of powerful and enormously dangerous hydroxyl radicals as well as singlet oxygen, both of which contribute to oxidative stress^18^. LRSL showed a significantly higher tendency to quench SOD radicals, as indicated by the dose dependent increase in percent inhibition (Fig. 1D). Furthermore, the hydroxyl radical is an extremely reactive ROS, which initiates auto-oxidation and attacks almost every biological molecule causes DNA damage, protein and lipids leading to mutagenesis, carcinogenesis and aging^28^. The current results are of great importance as LRSL exhibited great potential in scavenging the OH radicals (Fig. 1E).

FRAP and CUPRAC are important indicators of reducing potential of an antioxidant which is associated with the presence of compounds responsible for breaking the free radical chain through donation of hydrogen atom^29^. Since antioxidant capacity is directly correlated with their reducing capacity, both FRAP and CUPRAC assays provide a reliable method for the evaluation of antioxidant activities of various plant extracts and compounds^30^, our results are in conformity of these findings. The results demonstrated the noticeable antioxidant potential (in terms of FRAP and CUPRAC, measured as ascorbic acid equivalent) of LRSL, which was gradually increased with increasing concentrations of samples (Fig. 1F–G). Furthermore, the ORAC assay utilizes an AAPH-derived peroxyl radical that mimics lipid peroxyl radicals involved in the lipid peroxidation chain reaction *in vivo*. Inhibition of peroxyl radical-induced oxidation of a fluorescent probe, fluorescein, by antioxidants was serially monitored and the protective effect of an antioxidant is measured by assessing the area under the fluorescence decay curve (AUC)^22^. Net AUC values of Trolox and LRSL were increased in a dose-dependent manner (data not shown) and these results indicated that LRSL (6 μM) exerted similar antioxidant activity to 10 mM Trolox (Fig. 1H).

Cellular antioxidant capacity of antioxidants results from their direct capacity, defined as the capacity to quench free radicals, ROS and RNS either by donating hydrogen or electrons, whereas indirect capacity involves providing defense against oxidative stress through inducing the expression of phase II detoxifying and antioxidant enzymes^4^. ROS scavenging activity plays a pivotal role in cellular homeostasis during cell proliferation and maintenance. Several enzymes SOD, GPx, and CAT are associated with the removal of these free radical species within cells. If these enzymes are damaged by several occurrences of oxidative stress, degenerative diseases can result^31^. Cytosolic superoxide (O_2_^−^) is generated by the one-electron reduction of O_2_ through the slippage of electrons from the electron carriers of the mitochondrial electron transport chain. It is well known that O_2_^−^ is rapidly converted into H_2_O_2_ by SOD. Additionally, H_2_O_2_ can be detoxified H_2_O by the scavenging enzymes, GPx and CAT. These enzymes act together in the metabolic pathway of free radicals^26^,^32^. In this study, LRSL treatment significantly increased the both mRNA and protein levels of antioxidant enzymes such as SOD1, CAT and GPx-1 in RAW 264.7 cells (Fig. 3A,B), revealing the fact that LRSL have the ability to maintain the cellular homeostasis condition and to protect the cell from oxidative stress. These results were supported by the previous study^33^ which demonstrated that, LRSL treatment downregulated heat shock protein 27 (HSP27) in HepG2 cells, thereby potentially attenuating the oxidative stress condition.

Following the treatment of RAW 264.7 cells with LRSL for various times and with various doses, a significant increase in phase II enzymes at both the mRNA and protein levels were observed (Fig. 3A–C). HO-1 plays its antioxidant role by converting heme into the powerful pro-oxidant biliverdin and finally a strong antioxidant bilirubin^34^. Several nutrients with benefits related to oxidative stress, such as curcumin, caffeic acid ester, eckol, and hydroxytyrosol, have been reported to induce HO-1 expression^35^. Therefore, it is assumed that the induction of phase II enzymes might be another mechanism accounting for the benefit of LRSL against oxidative stress. To further investigate this mechanism in detail, first the mRNA and protein levels of Nrf2, the key regulator of phase II enzyme activation, was examined. Nrf2 is sequestered by the Kelch-like ECH-associated protein (Keap1) in the cytosol and constitutively targeted for poly-ubiquitination under a basal condition. Exposure of cells to electrophilic and oxidative stress, however, releases Nrf2 from Keap1, which facilitate the translocation of Nrf2 into nucleus, resulting in the activation of phase II cytoprotective gene^35^. In this study, inhibition of Nrf2 using a specific inhibitor, brusatol, it was found that the induction of HO-1 by LRSL was mitigated. This result confirmed previous conclusions regarding the regulation of phase II enzymes by Nrf2. Furthermore, Nrf2 protein levels were regulated by LRSL treatment. However, after 6 h, LRSL treatment promoted Nrf2 nuclear translocation, accompanied by decreased cyto-Nrf2 contents (Fig. 4F). Therefore, it is postulated that the mechanism of interaction between LRSL and Nrf2-Keap1 complex may mimic the action of other Nrf2 inducer such as 5-O-caffoylquinic acid modulates Nrf2 nuclear translocation and ARE-dependent gene expression such as HO-1, NQO-1, GST in HT29 cells^36^.

Other signaling pathways may involve in the regulation of phase II genes or interact with the Keap1/Nrf2/ARE system. The PI3K/AKT and MAPK pathways, including ERK, JNK and p38 activation, have also been suggested as upstream regulators of Nrf2^37^. In the current study, activation of MAPK pathways (ERK1/2, and p38) by LRSL was observed over time periods ranging from 15 to 360 min. To surprise, only p38 inhibitor, SB239063, efficiently blocked the expression of Nrf2 and HO-1 induced by LRSL. Ma *et al*.^33^ recently demonstrated that, LRSL treatment upregulated annexin A1, which can affect the formation and activity of protein complexes upstream of the MAPK signaling pathway in HepG2 cells, thereby contribute to the anticancer activity in liver cancer cell. Therefore, it is inferred that the Nrf2 activation induced by LRSL depends p38 activation pathway.

Generally, lignans exhibit a variety of biological properties including their antioxidant behavior^38^. The antioxidant mechanism of (+)-lariciresinol was proposed to be similar to that of secoisolariciresinol^39^ where the phenolic hydroxy functionalities at positions 4 and 4′ of two benzene rings act as principle trappers of free radicals.

## Conclusions

In this study, (+)-lariciresinol (LRSL) revealed significant and dose-dependent antioxidant potential in various *in vitro* models. Also, for the first time, the underlying mechanism, by which LRSL mitigate the oxidative stress through the transcriptional and translational regulation of phase I oxidoreductase and phase II detoxifying enzyme, was clarified. Further studies to confirm the *in vivo* efficacy of (+)-lariciresinol are warranted in an animal model.

## Additional Information

**How to cite this article:** Bajpai, V. K. *et al*. Antioxidant efficacy and the upregulation of Nrf2-mediated HO-1 expression by (+)-Lariciresinol, a lignan isolated from *Rubia philippinensis*, through the activation of p38. *Sci. Rep.* **7**, 46035; doi: 10.1038/srep46035 (2017).

**Publisher's note:** Springer Nature remains neutral with regard to jurisdictional claims in published maps and institutional affiliations.

## Supplementary Material

Supplementary Dataset 1

Supplementary Dataset 2

## Figures and Tables

**Table 1:** List of the primers used in this study.

SOD1	Forward	AAGCGGTGAACCAGTTGTGT
	Reverse	GCCAATGATGGAATGCTCTC
SOD2	Forward	AACTCAGGTCGCTCTTCAGC
	Reverse	CTGTAAGCGACCTTGCTCCT
GPx1	Forward	ACACCGAGATGAACGATCTG
	Reverse	ATGTACTTGGGGTCGGTCAT
Catalase	Forward	CACCCACGATATCACCAGATAC
	Reverse	GAAGACTCCAGAAGTCCCAGAC
HO-1	Forward	ACGCATATACCCGCTACCTG
	Reverse	TCCTCTGTCAGCATCACCTG
GCLC	Forward	GGAGGCGATGTTCTTGAGAC
	Reverse	GGGTGCTTGTTTATGGCTTC
GCLm	Forward	AGTTGCACAGCTGGACTCTG
	Reverse	TCGGGTCATTGTGAGTCAGT
NQO-1	Forward	CTGGCCCATTCAGAGAAGAC
	Reverse	GTCTGCAGCTTCCAGCTTCT
Nrf-2	Forward	ACATCCTTTGGAGGCAAGAC
	Reverse	GGGAATGTCTCTGCCAAAAG
	Reverse	TCGGGTCATTGTGAGTCAGT
GSTpi	Forward	GCCCAGATGGATATGGTGAA
	Reverse	ATGGGACGGTTCACATGTTC
