# Supplementary material for: Antioxidant efficacy and the upregulation of Nrf2-mediated HO-1 expression by (+)-lariciresinol, a lignan isolated from Rubia philippinensis, through the activation of p38
Source: Sci Rep. 2017 Apr 5;7:46035. doi: 10.1038/srep46035 (PMC5380954; doi:10.1038/srep46035)
Supplement: Supplementary Dataset 2 [file srep46035-s2.doc]

**Antioxidant efficacy and the upregulation of Nrf2-mediated HO-1 expression by (+)-lariciresinol, a lignan isolated from *Rubia philippinensis*, through the activation of p38**

**Vivek K. Bajpai^1,#^, Md Badrul Alam^2,#^, Khong Trong Quan^3^, Kyoo-Ri Kwon^2^, Mi-Kyoung Ju^2^, Hee-Jeong Choi^2^, Jong Sung Lee^4^, Jung-In Yoon^4^, Rajib Majumder^5,6^, Irfan A. Rather^1^, Kangmin Kim^7,*^, Sang-Han Lee^2,*^, MinKyun Na^3,*^**

^1^Department of Applied Microbiology and Biotechnology, School of Biotechnology, Yeungnam University, Gyeongsan, Gyeongbuk 38541, Korea

^2^Department of Food Science and Biotechnology, Graduate School, Kyungpook National University, Daegu 41566, Korea

^3^College of Pharmacy, Chungnam National University, Daejeon 34134, Korea.

^4^Kcellbio, Seoulsoop Kolon Digital Tower, Seongsuil-ro-4-gil, Seongdong-gu 04713, Seoul, Korea

^5^Department of Biological Sciences, Macquarie University, Sydney, NSW 2109, Australia

^6^Elizabeth Macarthur Agricultural Institute (EMAI), NSW Department of Primary Industries, Menangle, NSW 2567, Australia

^7^Division of Biotechnology, College of Environmental and Bioresource Sciences, Chonbuk National University, 79 Gobong-ro, Iksan-si-570-752, Jeonbuk, Republic of Korea

**^#^Both authors contributed equally to this work**

***Correspondence to:**

**Dr. Sang-Han Lee**; Email: [sang@knu.ac.kr](mailto:sang@knu.ac.kr); Tel: +82-53-950-7754

**Dr. Kangmin Kim**; E-mail: [activase@jbnu.ac.kr](mailto:activase@jbnu.ac.kr) Tel: +82-63-850-0834

**Dr. MinKyun Na**; E-mail: [mkna@cnu.ac.kr](mailto:mkna@cnu.ac.kr); Tel: +82-42-821-5925

**LSRL (50 µM**)


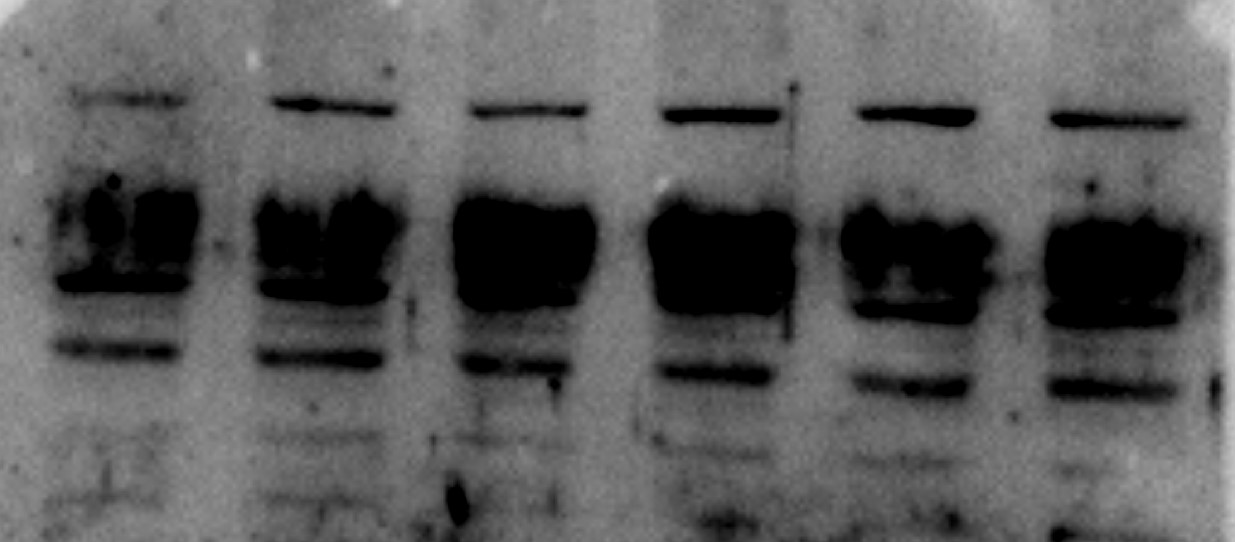

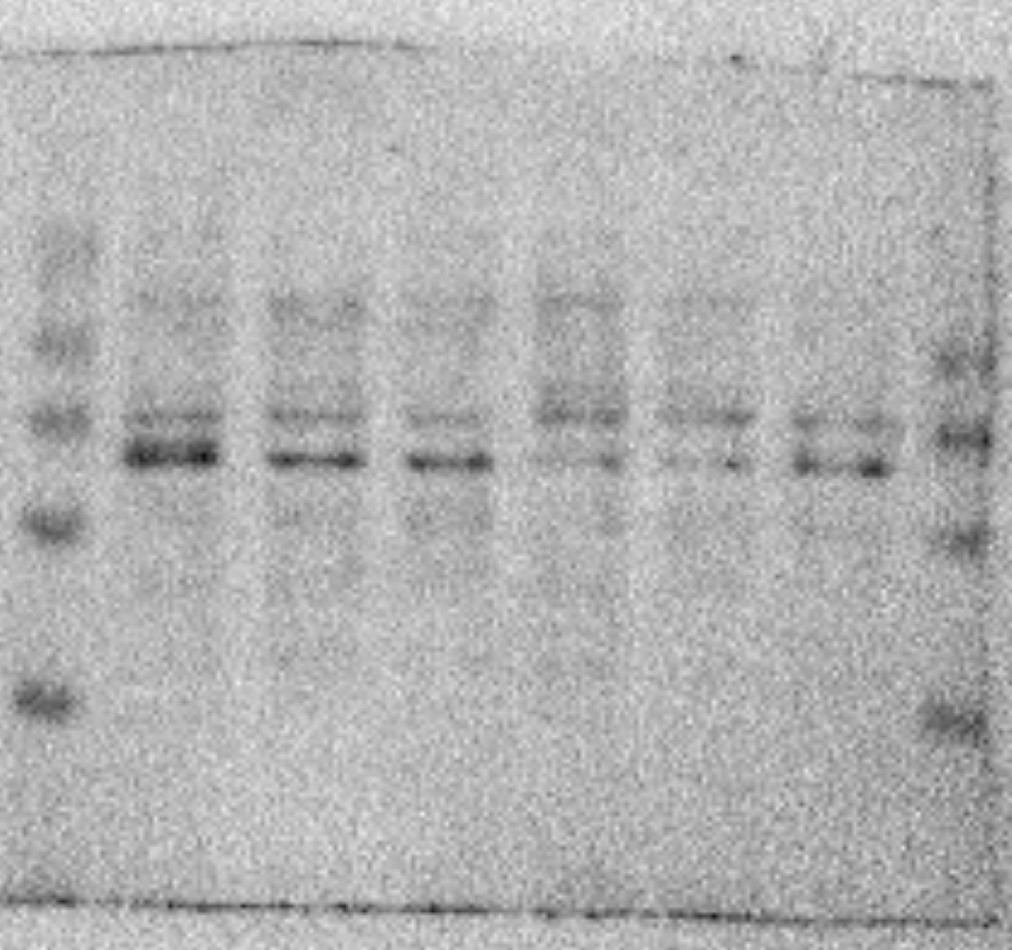
 0 15 30 60 180 360 (min)

**p-JNK**

**(p-T183/Y185)**

**Antibody reference (Bioworld technology, cataloge: BS4322)**

**46 KDa**

**46 KDa**

**JNK (Y185)**

**Antibody reference (Bioworld technology, cataloge: BS3631)**

[Type a quote from the document or the summary of an interesting point. You can position the text box anywhere in the document. Use the Drawing Tools tab to change the formatting of the pull quote text box.]

**Figure S4. The activation of JNK by LRSL.** RAW 264.7 cells were pretreated with LRSL for the indicated time and JNK kinase activation was analyzed by western blotting.
